# Overcoming Cancer Drug Resistance Utilizing PROTAC Technology

**DOI:** 10.3389/fcell.2022.872729

**Published:** 2022-04-25

**Authors:** Matthew R. Burke, Alexis R. Smith, Guangrong Zheng

**Affiliations:** Department of Medicinal Chemistry, College of Pharmacy, University of Florida, Gainesville, FL, United States

**Keywords:** proteolysis targeting chimera, targeted protein degradation, targeted therapy, cancer drug resistance, E3 ubiquitin ligase, drug discovery, acquired resistance

## Abstract

Cancer drug resistance presents a major barrier to continued successful treatment of malignancies. Current therapies inhibiting proteins indicated in cancer progression are consistently found to lose efficacy as a result of acquired drug resistance, often caused by mutated or overexpressed protein targets. By hijacking the cellular ubiquitin-proteasome protein degradation machinery, proteolysis-targeting chimeras (PROTACs) offer an alternative therapeutic modality to cancer treatments with various potential advantages. PROTACs specific for a number of known cancer targets have been developed in the last 5 years, which present new options for remission in patients with previously untreatable malignancies and provide a foundation for future-generation compounds. One notable advantage of PROTACs, supported by evidence from a number of recent studies, is that they can overcome some of the resistance mechanisms to traditional targeted therapies. More recently, some groups have begun researching the use of PROTACs to successfully degrade mutated targets conferring cancer resistance against first-line treatments. In this review, we focus on analyzing the developments in PROTACs geared towards cancer resistance and targets that confer it in the search for new and successful therapies.

## Introduction

Despite the dramatic progress in understanding cancer biology and cancer drug discovery in the last two decades, cancer drug resistance continues to be a major barrier to successful treatment of malignancies, as cancer cells gain new functions to overcome standard initial therapies (Ward et al., 2021). While many resistance mechanisms to cancer drugs exist, three of the major contributors render traditional small molecule therapies ineffective: drug inactivation, increased drug efflux, and target alteration (Housman et al., 2014). Of these, one of the most common pitfalls in drug discovery occurs when small molecule inhibitors (SMIs) become ineffective due to target protein alteration, including mutation and upregulation. Development of novel drugs has historically been the first line of defense against resistant cells, but their rates of discovery often lag in comparison to the rate of developing drug resistance. Researchers must continually develop new generations of therapies to keep up with the rate of modifications to the target proteins in cancer cells. Thus, alternative approaches to that strategy are needed to counter the emergence of drug resistance.

**FIGURE 1:**
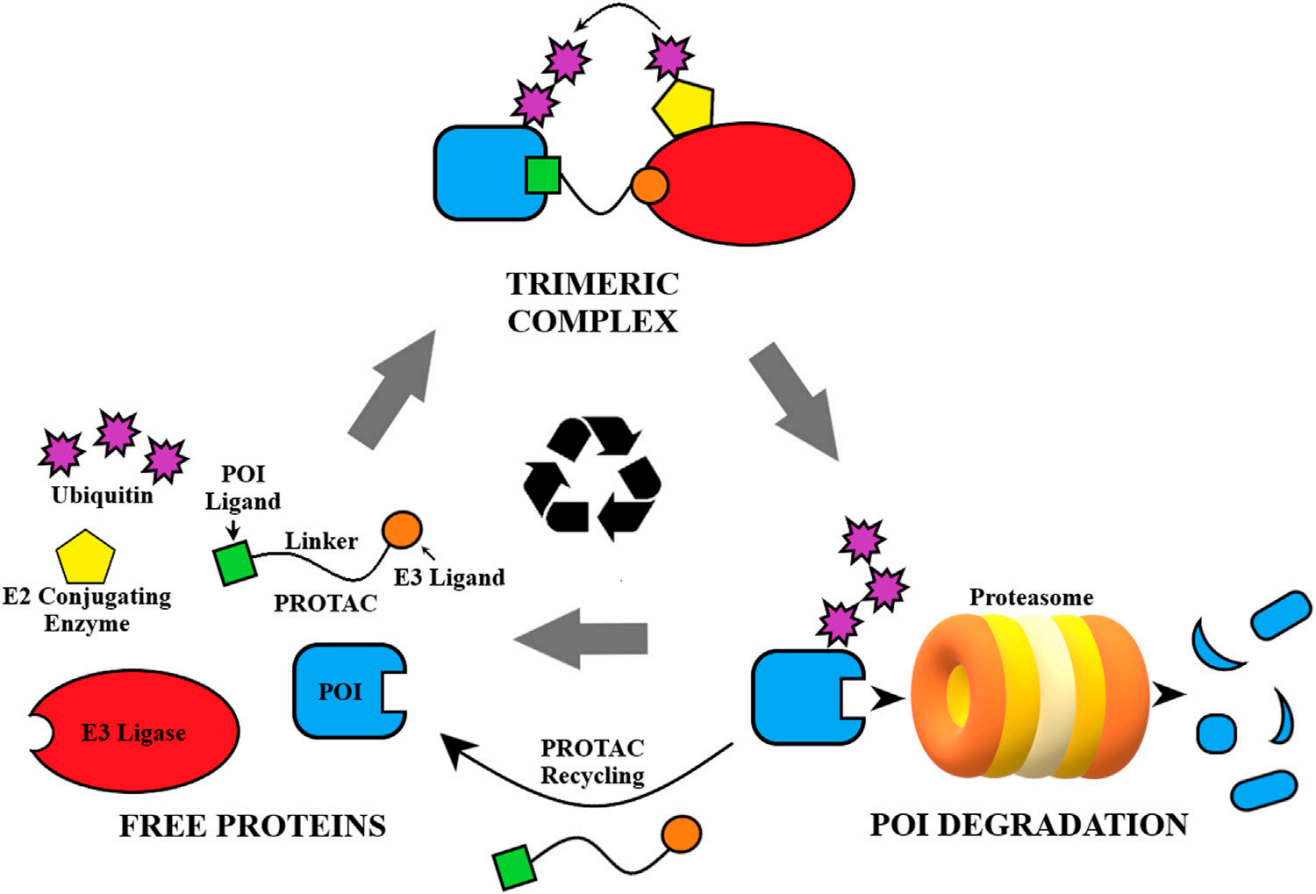
General depiction of the mechanism of action of PROTACs.

Proteolysis targeting chimera (PROTAC) technology presents a potent and promising alternative to traditional drug treatments, and has been a competitive drug discovery and research area in the last 5 years (Mullard, 2021; Garber, 2022). Using a heterobifunctional approach to recruit the ubiquitin-proteasome system (UPS) in the innate cellular protein degradation machinery, PROTACs are designed to induce proximity of an E3 ligase to the target protein of interest (POI) (Figure 1) (Sakamoto et al., 2001). The PROTAC binds to the POI as well as an E3 ligase and forms a ternary complex, allowing polyubiquitination of the target by an associated E2 ubiquitin-conjugating enzyme and subsequent destruction by the proteasome (Ciechanover et al., 2000; Lai and Crews, 2017). PROTACs are catalytic in nature and are able to eliminate the POI entirely in this process. For the cell to overcome this type of treatment it must resynthesize the POI, as opposed to quickly recovering from reversible SMIs (Churcher, 2018). Thus, the pharmacodynamic response to PROTACs could extend beyond the pharmacokinetic profile, driven by the synthesis rate of the POI (Mares et al., 2020). Additionally, PROTACs have been improving on typical treatments attained through SMIs with increased target selectivity (Jiang et al., 2019), greater potency/efficacy (Bondeson et al., 2015), and increased tissue selectivity (Khan et al., 2019). However, the biggest promise of this technology is its ability to target the traditionally “undruggable” proteome, i.e., proteins that lack a functional binding site, play a scaffolding role, or form aggregates, as PROTACs can be designed based on protein binders instead of functional ligands (Samarasinghe and Crews, 2021).

A number of proof-of-concept studies reported in literature have shown that PROTACs could be utilized in cases of resistant cancers where traditional inhibitors have failed to be effective. The catalytic mode of action renders PROTACs particularly effective in countering drug resistance due to overexpression of the POI. PROTACs can also be effective in drug resistance cases caused by point mutations because they can turn non-functional binders, loss-of-function binders, agonists, or weak inhibitors into potent degraders. Due to their ability for protein removal, PROTACs are particularly useful in resistance cases that are caused by scaffolding functions of the target proteins. In this review, we provide an overview of current progress in the development of PROTACs that have the potential of overcoming cancer drug resistance. In addition, we offer our perspectives on the potential cancer resistance mechanisms to PROTACs that may render them less effective.

## PROTACS Targeted Towards Treatment of Various Types of Drug Resistant Cancers

### BCR-ABL1-Targeted PROTACs to Address Drug Resistance in CML

**FIGURE 2:**
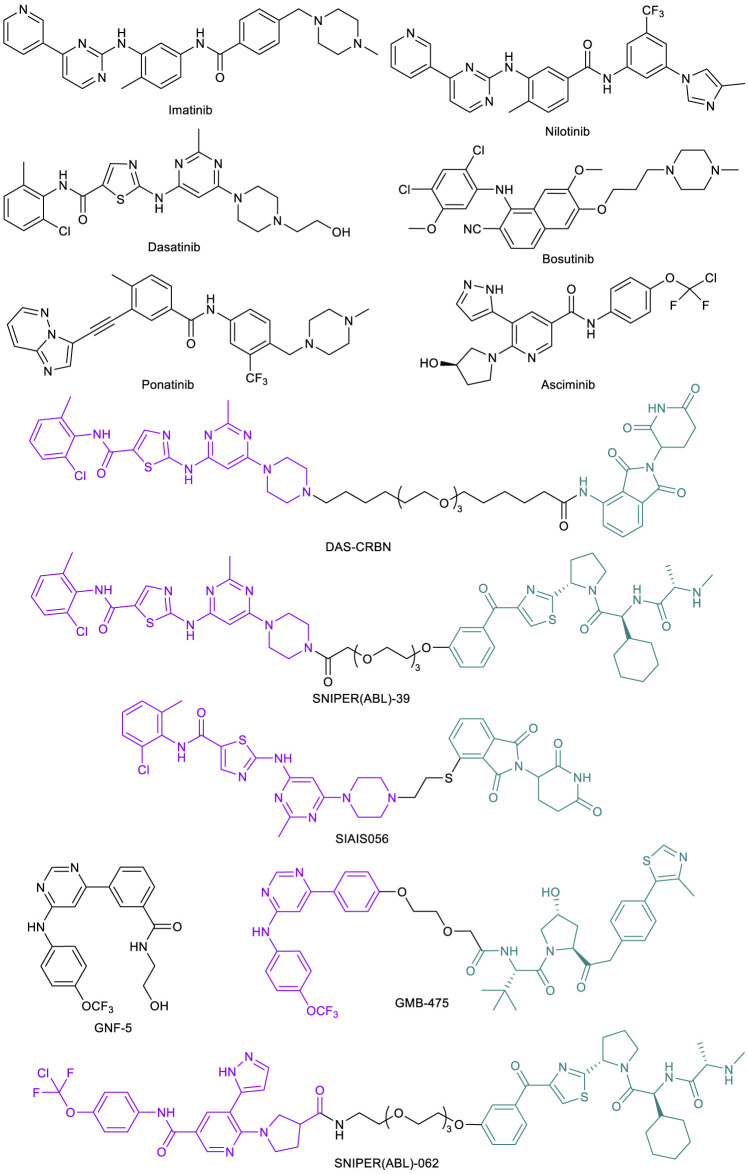
Key BCR-ABL1 small molecule inhibitors and BCR-ABL1-targeted PROTACs.

Nowell and Hungerford’s groundbreaking work in 1960 studying patients diagnosed with chronic myeloid leukemia (CML) led to the discovery of the cancer-causing Philadelphia (Ph) chromosome (Nowell and Hungerford, 1960). The Ph chromosome was later attributed to a genetic aberration and translocation between chromosomes 9 and 22, forming a fusion breakpoint cluster region (*bcr*) and Abelson tyrosine kinase (*abl*) fusion oncogene (Rowley, 1973). This oncogene product BCR-ABL1 is constitutively activated and a fundamental cause of CML (Ben-Neriah et al., 1986). Early work on ATP-competitive tyrosine kinase inhibitors (TKIs) led to the discovery of ABL inhibitor imatinib (Figure 2) (Druker et al., 1996). Initial studies also showed that in the presence of their compound, cell colony formation and growth was diminished by over 90% in BCR-ABL positive CML cells, leaving normal cells virtually unaffected. With the remarkable effect in preventing cancer progression and prolonging patient survival, imatinib gained FDA approval in 2001 (Druker et al., 2001, 2006). Imatinib was the first approved kinase inhibitor that transformed CML from a rapidly fatal disease to a manageable condition (Cohen et al., 2021). However, acquired drug resistance develops over time due to the extensive BCR-ABL1 mutations, which cause the reduced or abolished activity of imatinib. For example, in one study, it was found that seven point mutations (M244V, G250E, Y253F/H, E255K/V, T315I, M351T, and F359V) account for 85% of all resistance-associated mutations (Soverini et al., 2006). Next generation BCR-ABL1 ATP-competitive inhibitors including nilotinib (Blay and von Mehren, 2011), dasatinib (Das et al., 2006), bosutinib (Golas et al., 2003), and ponatinib (Huang et al., 2010) have been developed in response to the emergence of these imatinib-resistant mutations (Figure 2). Nilotinib, dasatinib, and bosutinib can inhibit many imatinib-resistant mutants but not the “gatekeeper” T315I mutant (Redaelli et al., 2009). Ponatinib is the only ATP-competitive BCR-ABL1 inhibitor that can effectively target against this gatekeeper mutant (Huang et al., 2010; O’Hare et al., 2009, 2012). However, due to the poor kinase selectivity, ponatinib may not be suitable for long term maintenance treatment. Asciminib (Figure 2) is a newly approved BCR-ABL1 inhibitor. Unlike the other inhibitors that bind to the ATP-binding site, asciminib binds to the allosteric myristate-binding site of BCR-ABL1 (Wylie et al., 2017; Schoepfer et al., 2018). Thus, asciminib has no overlapping resistance mutations with the ATP-competitive inhibitors, allowing the targeting of BCR-ABL1 mutants such as T315I.

Recent work on BCR-ABL1-targeted PROTACs provided a potential alternative option for patients showing drug resistance to these therapies. The Crews lab at Yale University initially designed a library of PROTACs with variations in POI ligand (imatinib, dasatinib, or bosutinib), linker, and E3 ligase ligand (for CRBN or VHL) (Lai et al., 2016). Among all these combinations, a dasatinib-based, CRBN-recruiting PROTAC, DAS-CRBN (Figure 2) showed the highest potency in degrading BCR-ABL1. Potent BCR-ABL1 degradation was also observed with SNIPER(ABL)-39 (Figure 2), a dasatinib-based, inhibitor of apoptosis protein (IAP)-recruiting PROTAC (also termed as SNIPER [specific and non-genetic IAP-dependent protein erasers]) (Shibata et al., 2017). Through an extensive structural optimization on the linker unit, Liu et al. (2021) developed a highly potent dasatinib-based, CRBN-recruiting BCR-ABL1 PROTAC SIAIS056 (Figure 2). This sulfur-substituted short carbon chain linker containing PROTAC exhibited a favorable *in vivo* pharmacokinetic profile and induced tumor regression in a tumor xenograft model. Importantly, SIAIS056 demonstrated degradation of all eight clinically relevant imatinib-resistant and dasatinib-resistant BCR-ABL1 mutants tested. Not surprisingly, no degradation was observed with BCR-ABL1 harboring a T315I mutation, as the gatekeeper threonine to isoleucine mutation blocks the ATP-binding site (Liu et al., 2021).

In order to target BCR-ABL1 mutants that render ATP-competitive inhibitors ineffective, the Crews lab developed BCR-ABL1 PROTAC GMB-475 (Figure 2) (Burslem et al., 2019). This compound, comprised of an allosteric ABL1 inhibitor GNF-5 (Figure 2) (Zhang et al., 2010) and a VHL-recruiting ligand, demonstrated significant levels of ubiquitination and degradation of the fusion protein target and downstream effector inhibition in human CML K562 cells as well as murine Ba/F3 cells, both expressing the oncogenic protein. Importantly, GMB-475 effectively degraded both wild-type (WT) BCR-ABL1 and BCR-ABL1 with T315I mutation. It also re-sensitized resistant Ba/F3 BCR-ABL1 cells to imatinib inhibition. Furthermore, GMB-475 induced apoptosis of CML CD43+ cells, while having virtually no adverse effects on healthy CD43+ cells at the same concentration (Burslem et al., 2019). Similarly, taking advantage of the allosteric binding site of the BCR-ABL1 protein, the Naito lab developed novel IAP-recruiting PROTACs. Of all compounds synthesized, SNIPER(ABL)-062 (Figure 2) exhibited the most promising activity profile in a series of *in vitro* assays (Shimokawa et al., 2017). These studies illustrated the ability of PROTAC technology to overcome drug resistance caused by ABL-1 ATP-binding site mutations.

### AR-Targeted PROTACs to Address Drug Resistance to AR Antagonists

One earliest example of utilizing PROTACs to overcome cancer drug resistance is in the treatment of castration-resistant prostate cancer (CRPC). Prostate cancer (PC) is one of the leading causes of death in men in the United States, with predictions showing this statistic to only be increasing (Tan et al., 2015). One protein with particular importance in PC is the androgen receptor (AR). These receptors are found in various tissues of the body, but most prominently in the prostate and adrenal gland, and play a key role in human development (Gao et al., 2005). The AR is an intracellular transcription factor that, when bound by the androgens dihydrotestosterone or testosterone, promotes a cascade of interactions, resulting in the binding of AR to androgen response elements in the promoter regions of targeted genes (Gao et al., 2005; Tan et al., 2015). AR signaling was originally considered a target for PC because of the increased activity in these cells and its promoting role in cell proliferation, and it remains a vital target to this day for this disease.

Most PCs are dependent on androgens in the early stages of the disease. For these androgen-sensitive PCs, surgical or chemical castration stops the production and release of most circulating androgens and starves the tumor of the AR activity, resulting in reduced cancer growth (Heinlein and Chang, 2004). The same result is often achieved with antiandrogen therapies such as AR antagonists. Unfortunately, many patients eventually experience a reemergence of PC after initial therapies in the form of an androgen-independent tumor, known as CRPC (Feldman and Feldman, 2001). One pathway for this resistance development is that the AR could become overexpressed or mutated to have more sensitivity to its ligands, allowing stimulated activity from even a reduced concentration of endogenous androgens (Montgomery et al., 2008). Alternatively, the AR could develop mutations that allow atypical ligands such as estradiol and other steroidal hormones to bind and activate AR (Veldscholte et al., 1990), resulting in activity without the influence of any ligand binding (Balbas et al., 2013), or allow an alternate pathway of cell growth activation that circumvents the AR entirely (Feldman and Feldman, 2001). In all cases, changes in the receptivity of the AR and its function make tumors resist androgen depletion therapies, and restore cell growth.

**FIGURE 3:**
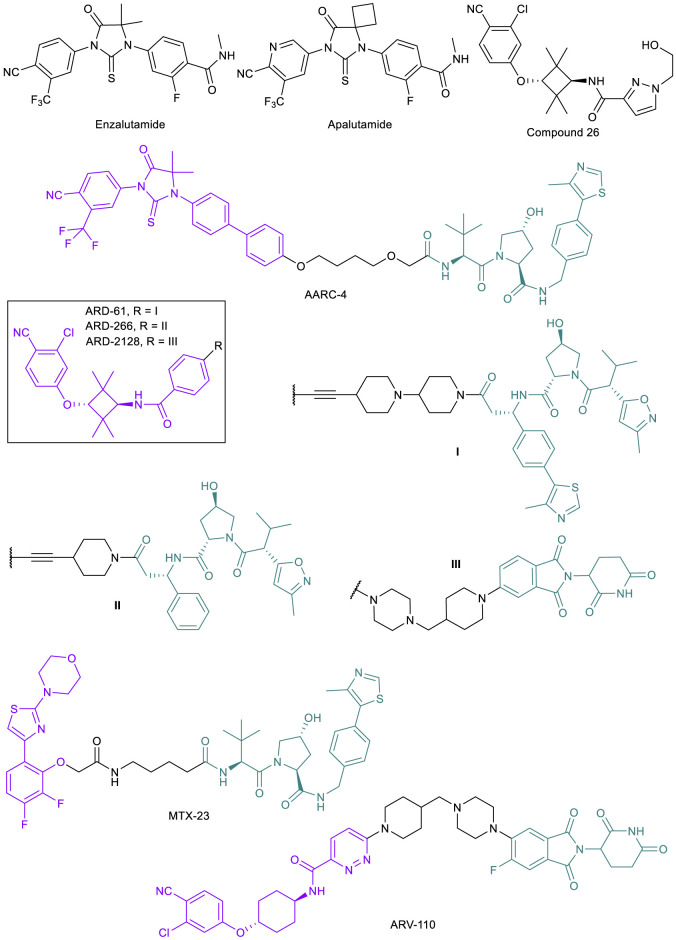
Key AR small molecule inhibitors and AR-targeted PROTACs.

Once the castration-resistant tumor emerges, the first line of defense against PC becomes ineffective. While new AR antagonists such as enzalutamide and apalutamide (Figure 3) continue to be designed as better androgen deprivation therapies, it is known that these drugs will only be clinically useful up to the point where resistance arises (Balbas et al., 2013). These mutations are often a result of the antiandrogen therapy itself; for example, prolonged exposure of PC cell lines and tumors to enzalutamide has been shown to result in the emergence of the F876L point mutation in the AR ligand binding domain (LBD) where enzalutamide binds in every strongly resistant mutated cell line (Balbas et al., 2013; Korpal et al., 2013). Along with other point mutations such as T878A, F876L results in a switch from antagonist to agonist activity of enzalutamide, allowing for cancer cells to continue proliferation (Balbas et al., 2013; Gustafson et al., 2015). Other drug resistance mechanisms that can be attributed to the progression to CRPC include AR upregulation and expression of AR splice variants (AR-SVs) lacking the LBD, upregulation of coactivators, increased androgen synthesis, and activation of other signaling pathways that are related to AR reactivation (Chism et al., 2014). There are limited treatment options once the cancer has progressed to CRPC.

The emergence of PROTAC technology, however, has raised the hope of finding new treatments for CRPC. As mentioned above, the majority of potential resistance mechanisms in CRPC still involve the presence of the AR, and thus utilizing PROTACs to degrade AR, AR mutants, or AR-SVs could be effective (Kargbo, 2019). AR-degrading PROTACs are typically derived from the existing libraries of potent AR antagonists, and modified with optimization of the linker unit and alteration of the binding moiety to different E3 ligases. The effectiveness of PROTACs for treating CRPC has been demonstrated in multiple cases, originating from both academic laboratories and industrial settings. While publications involving AR targeted PROTACs began appearing in the 2000s (Sakamoto et al., 2003; Schneekloth et al., 2008; Tang et al., 2009), only recently have PROTACs been developed specifically in response to resistant tumors. In 2018, the Crews lab reported ARCC-4 (Figure 3), a VHL-recruiting enzalutamide-based PROTAC (Salami et al., 2018). After observing AR degradation in VCap and LNCaP cells with ARCC-4 treatment, they engineered LNCaP cells to overexpress the F876L mutation clinically relevant in PC patients that stop responding to enzalutamide treatments. They found that by degrading the AR, ARCC-4 decreased the agonist activity typically seen with antagonist treatments post-resistance acquisition (Kregal et al., 2020). They also tested several other mutations using engineered HEK293T cells and found that ARCC-4 degraded all of the AR mutants. The Wang lab at the University of Michigan reported a series of highly potent VHL-recruiting AR-PROTACs, such as ARD-61 and ARD-266 (Figure 3) (Han et al., 2019a; Han et al., 2019b; Kregal et al., 2020), based on compound 26 (Figure 3) (Guo et al., 2011). Since the publication of the initial discovery of ARD-61, the authors continued their research and have published an additional study where the PROTAC was tested specifically against enzalutamide-resistant (Enz^R^) cell lines with great success compared to the inhibitor alone, resulting in an IC_50_ of 472 nM against the aggressive CWR-R1 Enz^R^ cell line and similar effects in other CRPC lines (Kregal et al., 2020). After the success of their previous compounds, the Wang lab developed a CRBN-recruiting AR-PROTAC ARD-2128 (Figure 3) with good oral bioavailability (67% in mice) (Han et al., 2021). However, the AR antagonists-derived PROTACs will not be effective in CRPC with AR-SVs such as AR-V7 that lack the LBD. To this end, MTX-23 (Figure 3), a VHL-recruiting AR-PROTAC based on a ligand targeting the DNA binding domain (DBD), has been developed to induce degradation of AR-V7 (Lee et al., 2021). MTX-23 is effective in inhibiting cell growth in multiple resistance PC cell lines and shows efficacy in tumor models.

Development of AR-PROTACs for PC treatment has been highly active in industry. Arvinas’ ARV-110 (Figure 3) was the first PROTAC to make it to clinical trials and is currently in Phase II (NCT03888612) (Halford, 2021; Mullard, 2021). ARV-110 is an orally bioavailable AR degrader for the treatment of metastatic CRPC in men. The drug displays high efficacy in inhibiting tumor growth in an enzalutamide-insensitive patient-derived xenograft (PDX) model (Snyder et al., 2021 presented). Based on Phase I data, ARV-110 is well-tolerated and induces 70–90% AR degradation in biopsies from one patient. However, as predicted, patients with point mutations such as L720H that render ARV-110 ineffective in degradation or with AR-SVs (AR-V7) did not respond to the drug. ARV-766 (NCT05067140) and CC-94676 (NCT04428788) are two AR-PROTACs that began Phase I trials recently, developed by Arvinas and Celgene, respectively. Both drugs are being tested in men with metastatic CRPC.

### ER-Targeted PROTACs to Address Drug Resistance to SERMs

**FIGURE 4:**
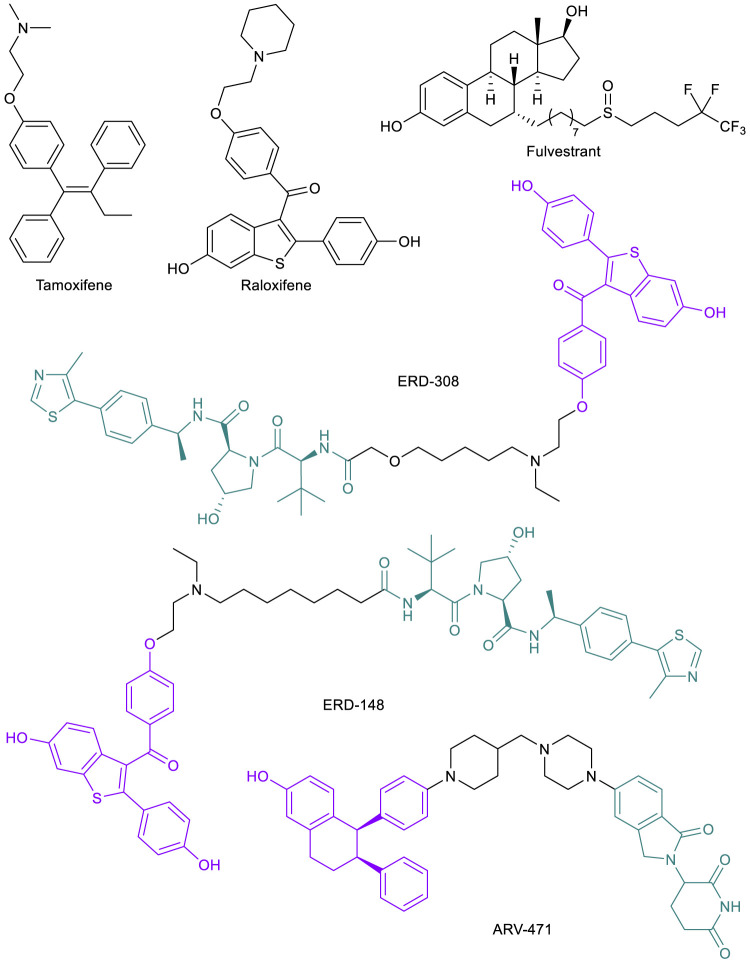
Key ER small molecule inhibitors and ER-targeted PROTACs.

In a similar pattern to AR-targeted PROTAC development, the estrogen receptor (ER) has become a target for the PROTAC technology due to its influence in breast cancer (BC) progression and metastasis (Sakamoto, 2005). BC has become the most commonly diagnosed malignancy worldwide (Sung et al., 2021). The cancer is largely ER*α* (the major controller of estrogen signaling over ER*β*) positive (ER^+^), which has been found to have vulnerability to endocrine therapies including selective ER modulators (SERMs) such as tamoxifen and raloxifene that directly alter ER activities (Figure 4). Although SERMs continue to play important role in treating BC, several resistance mechanisms toward these drugs have been observed, including hypersensitivity of ER*α* to estrogen and ligand independent or SERM-stimulated activation of ER*α*, driven by mutations in the LBD or stabilization of ER*α* (Hanker et al., 2020). However, these endocrine resistant tumors still largely depend on ER*α* signaling. Thus, the strategy to combat this mechanism of resistance was to eliminate ERα expression, which led to the discovery of a new class of drugs called selective ER degraders (SERDs). Currently, fulvestrant (Figure 4) is the only SERD approved by the FDA, but numerous new compounds are under clinical development (Hernando et al., 2021). In both animal models and clinical settings, SERDs have proven to be effective in countering several resistance mechanisms to SERMs (Lu and Liu, 2020). SERDs bind to the ER*α* and appear to induce a conformational change of the protein that not only inhibits ER*α* function, but also causes its degradation by the proteasome. The mechanism of SERD-induced ER*α* degradation is not well understood but could be due to the protein conformational change resulting in exposure of hydrophobic surfaces (Pike et al., 2001).

ER*α-*targeted PROTACs offer alternative approaches to eliminate the protein. Compared to SERDs, PROTAC-induced ER*α* degradation is more complete and more potent, and thus could be more effective in treating endocrine resistant BC. In addition, ER*α-*PROTACs have a clear mechanism of action in inducing protein degradation, which makes the drug development path more straightforward than SERDs. Following the first ER*α*-PROTAC in 2003 (Sakamoto et al., 2003), many strides have been made by academic researchers and industrial laboratories (Wang et al., 2018; Lin et al., 2020). For one example, the Wang lab published their development of several highly potent VHL-recruiting raloxifene-based ER*α*-PROTACs, the most potent one being ERD-308 (Figure 4), with a DC_50_ of 0.17 and 0.43 nM in two BC cell lines (Hu et al., 2019). Compared to fulvestrant, ER*α* degradation induced by ERD-308 was more complete and the compound also displayed a stronger inhibition of cell proliferation in MCF-7 cells (Hu et al., 2019). In a follow-up study, ERD-148 (Figure 4), a close analog of ERD-308, potently degraded ER*α* in both WT MCF-7 cells and MCF-7 cells harboring Y537S or D538G mutations (Gonzalez et al., 2020). The most advanced ER*α*-PROTAC is ARV-471 (Figure 4), an orally bioavailable CRBN-recruiting PROTAC developed by Arvinas for the treatment of patients with locally advanced or metastatic ER^+^/HER2^-^ BC. ARV-471 is currently in Phase II clinical trials (NCT04072952) (Halford, 2021). In a PDX model with a Y537S mutation, ARV-471 displayed high efficacy in tumor growth inhibition, while fulvestrant only had moderate effect (Flanagan et al., 2019). ARV-471 is well tolerated based on Phase I data (Lin et al., 2020). At their most recent related news update, Arvinas reported up to 89% ER degradation observed in biopsies from 14 patients with doses up to 500 mg daily, with a mean degradation of 64% (Arvinas 2021). The success of these compounds to this point has inspired additional development and patent applications and will presumably remain a groundbreaking area of research and drug development in the future.

### BET-Targeted PROTACs to Address Drug Resistance in CRPC and TNBC

**FIGURE 5:**
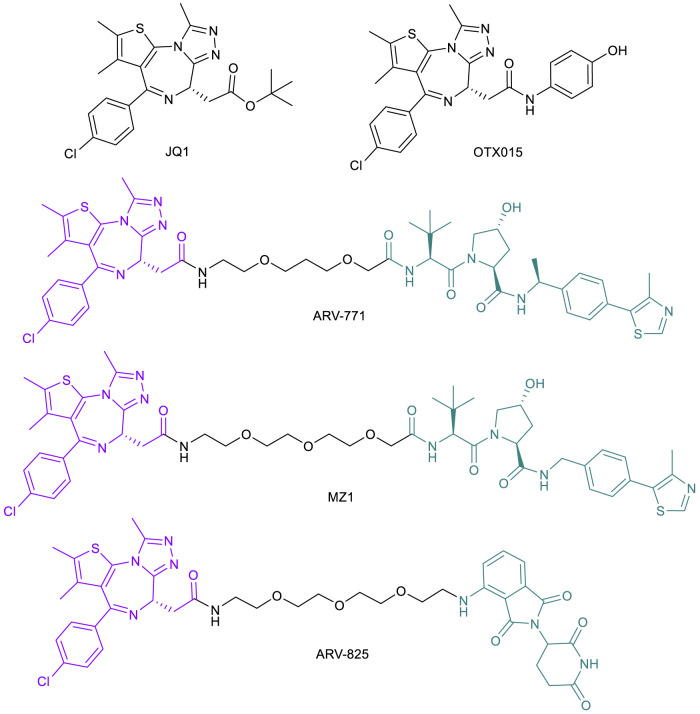
Key BET protein small molecule inhibitors and BET-targeted PROTACs.

Continuing research into novel treatments for metastatic CRPC with *de novo* or acquired resistance to AR suppression treatment, the bromodomain and extra terminal (BET) protein family (BRD2, BRD3 and BRD4) is another target considered to improve therapy. As previously discussed, androgen deprivation therapies can initially lead to remission in stage I or II (Trewartha and Carter, 2013) but eventual progression may occur, at which point the cancer is classified as CRPC, bringing a worse prognosis and an average survival time of 16–18 months (Amaral et al., 2012). BRD4 binds ARs directly and influences the progression of CRPC, but the interaction can be blocked by the pan-BET inhibitor JQ1 (Figure 5), resulting in a cellular inability to transcribe AR-mediated oncogenes (Asangani et al., 2014). In addition, recent studies indicate that BRD4, not BRD2 or BRD3, regulates key epithelial to mesenchymal transition (EMT) driver genes in CRPC (Shafran et al., 2021). Many BET-targeting PROTACs have been reported (Yang et al., 2019) after initial results showed that PROTACs based on a BRD4 ligand linked to a CRBN ligand led to rapid and sustained degradation of the BET family member proteins in cancer cells (Lu et al., 2015).

Given the ability of CRPC cells to confer secondary resistance even after targeting AR signaling pathways, applying PROTAC technology may be a more effective treatment with extended remission results. Initial exploration into the therapeutic potential of BET-targeted PROTACs for CRPC was based on ARV-771 (Figure 5), a VHL-recruiting JQ1-based pan-BET degrader (Raina et al., 2016). ARV-771 exhibited potent BRD2/3/4 degradation and higher potency than JQ1 and OTX015 (another pan-BET inhibitor; Figure 5) (Raina et al., 2016) in suppressing BET function, as indicated by the depleted downstream effector c-MYC protein in several cell line models of CRPC that are resistant to enzalutamide. In these cell lines, ARV-771 is 10- to 500-fold more potent than JQ1 in inhibiting cell proliferation. In addition, ARV-771 induces apoptosis in CRPC cells *in vitro*, whereas JQ1 and OTX015 have only a cytostatic effect, which further demonstrated the superiority of BET-PROTACs compared with BET inhibitors in treating CRPC. These results have been consistent in xenograft models of CRPC, with downregulation of BRD4 and downstream effector c-MYC, as well as tumor regression. Notably, two of 10 mice showed no appreciable tumor mass and no significant loss in body weight, indicating minimal systemic toxicity (Raina et al., 2016). These promising results offer potential for greater incidence of and longer duration of remission in men diagnosed with late stage CRPC.

BET-PROTACs have also found applications in countering drug resistance in triple negative breast cancer (TNBC), a highly aggressive tumor that accounts for 15% of all BCs (Lehmann et al., 2011). The BRD4-c-Myc axis appears to drive these tumor growths and was found to be targetable by BET inhibitors such as JQ1 (Ocaña and Pandiella, 2017; Ocaña et al., 2017). However, prolonged treatment with BET inhibitors can result in acquired resistance. BRD4 overexpression is indicated as one of the many potential resistance mechanisms to BET inhibitors, which can be effectively countered by a BET-PROTAC (Noblejas-López et al., 2019). Indeed, BET-PROTACs MZ1 and ARV-825 (Figure 5) have been shown to efficiently degrade BRD4 in both an MDA-MB-231 TNBC cell line and a JQ1-resistant cell line MDA-MB-231R (Noblejas-López et al., 2019). The BET-PROTACs also showed an antiproliferative effect in both cell lines with a profound effect on caspase-dependent apoptosis. Importantly, MZ1 was also active in inhibiting MDA-MB-231R growth in a xenograft model, suggesting the clinical potential of BET-PROTACs in treating TNBC (Noblejas-López et al., 2019).

### CDK4/6-Targeted PROTACs to Address Drug Resistance Caused by CDK6 Overexpression

**FIGURE 6:**
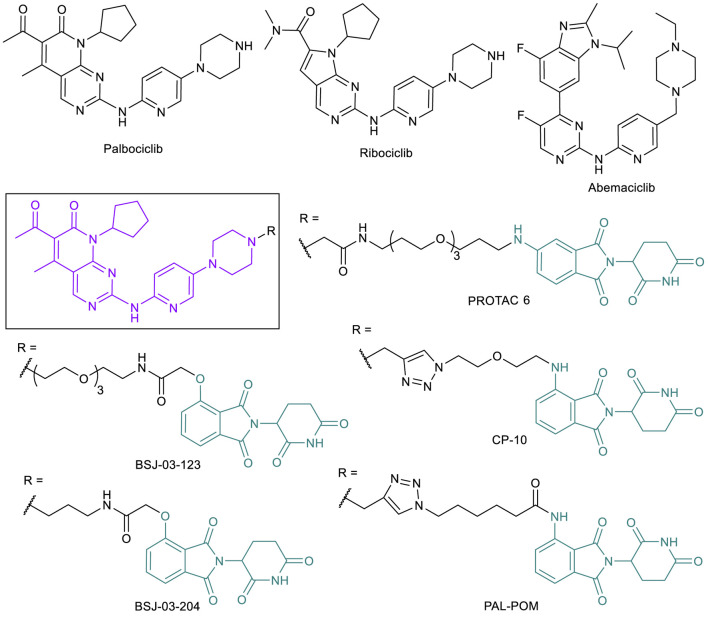
Key CDK4/6 small molecule inhibitors and CDK4/6-targeted PROTACs.

Since the discovery of cyclin-dependent kinase (CDK) function as regulators in cell division (Nurse, 2002; Sherr, 1996), CDK inhibitors have been extensively evaluated as cancer treatments (Zhang et al., 2021). CDK1 (regulator of mitosis), CDK2, CKD4, and CDK6 (regulators of the S phase) all play a role in proliferative signaling to proceed through the cell cycle when associated with various necessary cyclins (Serrano, et al., 1993; Asghar et al., 2015). Other CDKs, such as CDK7, CDK8, and CDK9, are involved specifically with transcription regulation, and play a different yet still important role in the cell (Asghar et al., 2015). While early CDK inhibitors did not make it through clinical trials due to indiscriminate binding, more selective CDKIs have since been found to have less cytotoxic effects and increased potency. CDK4 interacts with cyclin D and plays an essential role in both breast tumor initiation and proliferation (Yu et al., 2006). Inhibiting the activity of CDK4/6 reduces phosphorylation activity, leading to reduced tumor formation and growth. After the observations that pan-CDK inhibitors were not viable treatments due to toxicity, researchers began searching for selective inhibitors, and often selected CDK4 or combined CDK4/6 in response to the importance of the kinases in BC. Following the first FDA approved CDK4/6 inhibitor palbociclib (2015), two more CDK4/6 inhibitors, ribociclib (2017) and abemaciclib (2017), have been approved (Figure 6) and many more are currently in clinical trials (Sanchez-Martinez et al., 2015, 2019). CDK4/6 inhibitors are found to be most effective in BC combination therapies, and all three drugs are approved for this malignancy (Yuan et al., 2020). These inhibitors have proven to be effective, but a combination of the need to counter acquired resistance and the desire to also target the kinase-independent functions of CDK4/6 has made PROTAC technology an appealing direction in this field (Li et al., 2018; Rana et al., 2019). Resistance occurs through different pathways in this system, typically because of CDK6 overexpression caused by CDK6 mutation-triggered amplification (Yang et al., 2017; Knudsen and Witkiewicz, 2017) or loss of a tumor suppressor protein like FAT1 or RB1 (Li et al., 2018). Thus, CDK4/6 targeted PROTACs can be effective in combating drug resistance caused by CDK6 overexpression. Both CDK6 selective degraders like BSJ-03-123, PROTAC 6, and CP-10 (Brand et al., 2019; Rana et al., 2019; Su et al., 2019; Anderson et al., 2020) and CDK4/6 dual degraders such as BSJ-03-204 and PAL-POM (Jiang et al., 2019; Zhao and Burgess, 2019) have been reported (Figure 6). These PROTACs have typically used combinations of VHL or CRBN E3 ligase ligands and FDA-approved CDK4/6 inhibitors to achieve degradation, with variations in linker lengths, connection points, and functional groups. Many of these PROTACs have proved to be potent enough in early stage examination to have potential in clinical studies (Su et al., 2019; Zhao and Burgess, 2019; Anderson et al., 2020). CP-10, a CRBN-recruiting palbociclib-based CDK6 selective PROTAC, has been shown to effectively degrade both WT and mutated and overexpressed CDK6, supporting the potential of applying CDK4/6-PROTACs to overcome palbociclib resistance.

### EGFR-Targeted PROTACs to Address Resistance Caused by Point Mutations and Scaffolding Function

**FIGURE 7:**
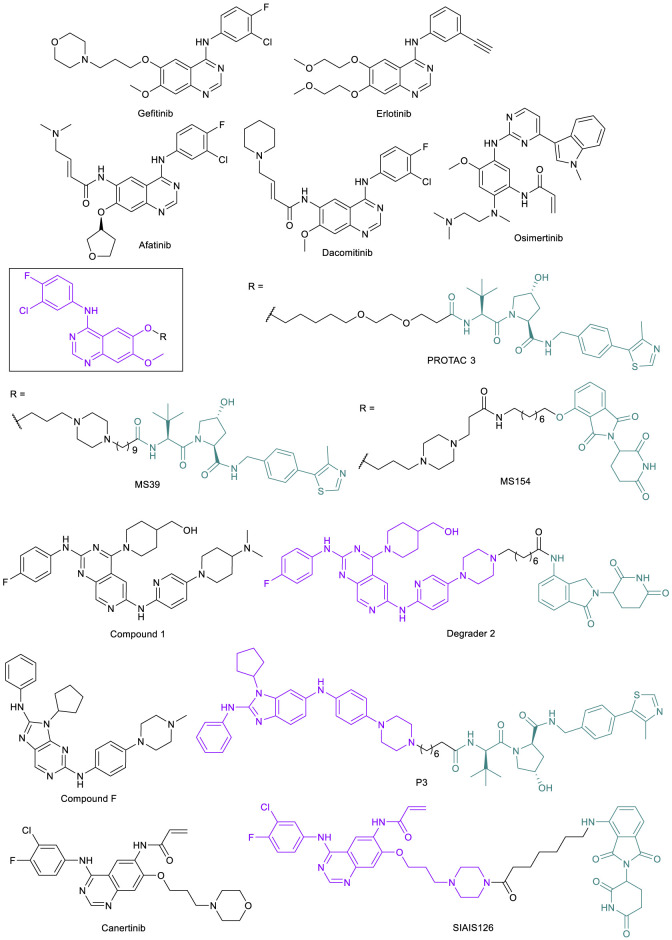
Key EGFR small molecule inhibitors and EGFR-targeted PROTACs.

The epidermal growth factor receptor (EGFR) is a single transmembrane-spanning protein that promotes downstream pro-proliferation and survival signaling in the cell, activated through C-terminal phosphorylation by the EGFR tyrosine kinase domain (Lynch et al., 2004). The EGFR has been a potential target to treat non-small cell lung cancer (NSCLC) since the discovery that it is often overexpressed in lung cancer tissue compared to surrounding normal tissue (Rusch et al., 1993). The first EGFR TKI, gefitinib, was approved by the FDA for the treatment of NSCLC in 2003, followed by erlotinib in 2004 (Figure 7). While the percentage of patients who were effectively treated by gefitinib was small (for example from one clinical study, 27.4% for Japanese patients and 10.4% for patients of European descent) (Paez et al., 2004), those who showed a response had dramatic improvements to their condition, enough to warrant further study into the cause for variability in treatment success. It was found that there were several mutation types that were common among most cases of gefitinib success, most commonly including the L858R point mutation and deletions in exon 19 (Ex19del) that typically overlapped to include codons 747-750, along with a few others (Lynch et al., 2004; Paez et al., 2004). L858R and Ex19del are activating mutations in EGFR that act as oncogenic drivers in NSCLC (Sharma et al., 2007; Cheng et al., 2020). *In vitro* studies show that the L858R mutation decreases the binding affinity of ATP to EGFR (Yun et al., 2008), leading to the increased potency of “first-generation” EGFR TKIs like gefitinib and erlotinib that bind to the ATP binding site. Thus, gefitinib and erlotinib were approved by the FDA for the treatment of NSCLC patients with these activating mutations in EGFR.

However, even as the success of the first-generation EGFR TKIs was proven for the population of NSCLC patients with these activating mutations in EGFR, resistance to these drugs quickly developed. In one of the original examples of resistance, a patient who had responded to gefitinib to the point of complete remission relapsed after 2 years; an analysis of the biopsy discovered a mutation of gatekeeper threonine 790 to methionine (T790M) (Kobayashi et al., 2005). The acquired mutation has since been found to be the reason for resistance in many cases of NSCLC that are treated with first-generation EGFR TKIs, as this mutation increases the affinity of the protein for ATP (Yun et al., 2008). To overcome this resistance mechanism, a new class of compounds was created by introducing an irreversible covalent bond-forming warhead that alkylates key residue cysteine 797 (C797) and was effective in both activated EGFR and EGFR^T790M^ (Zhou et al., 2009). The activity of these new drugs including afatinib and dacomitinib (Figure 7) is heavily dependent on C797, as it is the reactive residue for covalent binding, but it was soon found that a C797S mutation greatly reduced potency by a 100-fold difference in IC_50_ (Godin-Heymann et al., 2008; Zhou et al., 2009; Oxnard et al., 2018). After treatment of EGFR^T790M^ mutants with the covalent inhibitor AZD9291 (later named osimertinib) (Figure 7), Thress et al. (2015) demonstrated that C797S was one of the predominant resulting mutations observed in 40% of 15 patients who experienced progression while taking the drug.

The C797S mutation is thus a resistance mechanism to the third-generation FDA-approved drug osimertinib, designed to target both activated EGFR and EGFR^T790M^ with selectivity over wild type EGFR (EGFR^WT^) (Oxnard et al., 2018). Current EGFR TKI drug discovery has been focusing on reversible inhibitors that are effective against the triple mutation (activated EGFR through L858R or Ex19del, T790M, and C797S); some have shown promise in selectivity for mutants over EGFR^WT^ or occupying both the ATP binding site and the allosteric site on the enzyme (Shen et al., 2019; Li et al., 2019). The early success of these compounds, however, does not minimize the fact that EGFR mutates frequently with inhibitor therapies and there is a need to find better strategies. The development of PROTACs to degrade various forms of EGFR is a potential approach to counter the resistance mutations from EGFR TKI treatment. The first EGFR-targeted PROTACs such as PROTAC 3 (Figure 7) were published in 2018 by the Crews lab, and were created based on gefitinib and a few other known inhibitors linked to a VHL ligand (Burslem et al., 2018). The fact that the transmembrane EGFR protein can be degraded by PROTACs was a breakthrough in the field.

EGFR also has non-enzymatic functions, acting as a binding partner for other receptor tyrosine kinases (RTKs), that are independent of its kinase activity. The interaction can activate other RTKs to induce kinome rewiring, which contributes to resistance against EGFR TKIs (Jo et al., 2000; Stuhlmiller et al., 2015). Thus, the degradation of EGFR also removes the scaffolding functions of the target and increases the effective time as the protein must be resynthesized before signaling can resume (Burslem et al., 2018; Cheng et al., 2020). Based on gefitinib, the Jin lab improved on the potency of PROTAC 3 with their own VHL-recruiting EGFR-PROTAC MS39, and also created a CRBN-recruiting EGFR-PROTAC MS154 (Figure 7) (Cheng et al., 2020). However, while these compounds had DC_50_ values at or below 25 nM in activated EGFR cell lines, both of these compounds were based on gefitinib and so were ineffective against mutants that had developed resistance through the T790M mutation.

The Zhang lab at Xi’an Jiaotong University made significant strides in development of PROTACs targeting the mutated EGFR. Their EGFR-PROTACs were based on a fourth-generation EGFR TKI compound 1 (Figure 7), which they discovered in the lab with potent inhibitory activity against EGFR^L858R/T790M/C797S^ (Zhang et al., 2020). However, one of the most potent PROTACs, degrader **2** (Figure 7), was potent in degrading EGFR^Ex19del^ (DC_50_ < 50 nM) but only weakly degraded EGFR^L858R/T790M^ in H1975 cell line. A later publication from the same lab reported PROTAC P3, based on a different EGFR TKI compound F (Yang et al., 2012) that has activity against both EGFR^Ex19del^ and EGFR^L858R/T790M^ (Figure 7). P3 demonstrated its activity against both the activated EGFR and the double mutant, seen with its activity against the HCC827 (DC_50_ = 0.51 nM) and H1975 (DC_50_ = 126.2 nM) cell lines, respectively (Zhao et al., 2020). Most recently, the CRBN-recruiting EGFR^L858R/T790M^ degrader SIAIS126 (Figure 7) was reported, based on an irreversible EGFR inhibitor canertinib (Figure 7) (Qu et al., 2021). SIAIS126 selectively degraded mutated EGFR but not EGFR^WT^ in A549 cell line. While this does not exhaustively show every PROTAC created targeting EGFR or various mutations, there are still at this point no published PROTACs that can degrade EGFR^C797S^. With the emergence of more EGFR TKIs that target the triple mutation EGFR^L858R/T790M/C797S^, however, the potential is there for new PROTAC development that may one day be able to target these resistant cells.

### BRAF-Targeted PROTACs to Address Drug Resistance Caused by Target Mutation

BRAF belongs to the rapidly accelerated fibrosarcoma (RAF) family of serine/threonine protein kinases. Along with two other members of the RAF family, ARAF and CRAF, BRAF contributes to major cellular functions by transducing downstream signals of RAS to the mitogen-activated protein kinase (MAPK) cascade. While mutations in ARAF or CRAF are very rare in cancer cells, BRAF is one of the most frequently mutated protein kinases and mutations in BRAF account for ∼8% of all cancer cases (Fransén et al., 2004; Garnett and Marais, 2004; Emuss et al., 2005; Lee et al., 2005). The BRAF^V600E^ is the most prevalent among all mutations, and has been shown to make BRAF constitutively active, resulting in activation of downstream oncogenesis signaling pathways (Tsai et al., 2008). The most common cancer associated with BRAF mutations is melanoma, with 59% of cell lines analyzed having some kind of polymorphism. This is followed by colorectal cancers (18%), liver cancers (14%), and gliomas (11%) (Davies et al., 2002).

**FIGURE 8:**
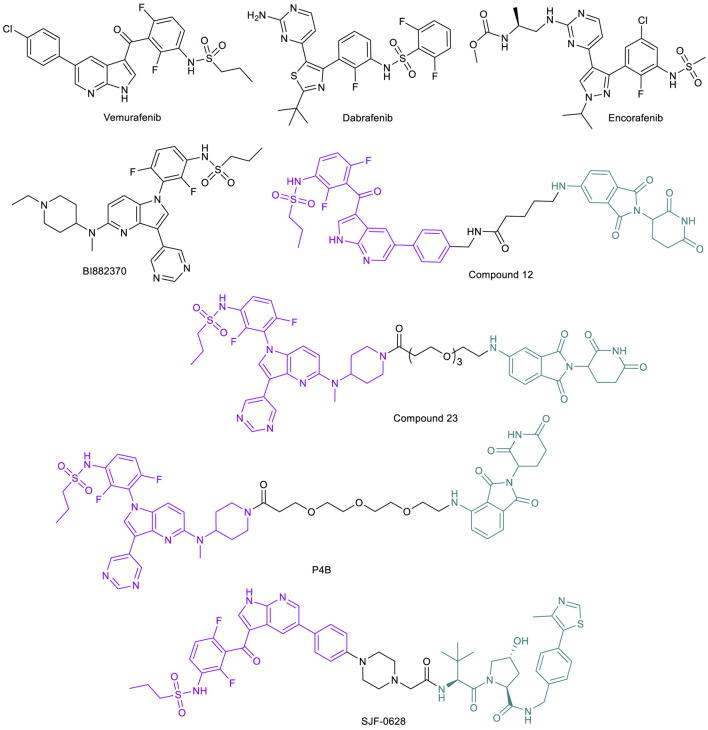
Key BRAF kinase small molecule inhibitors and BRAF-targeted PROTACs.

Given the significance of the BRAF^V600E^ mutant and its activity in various cancers, the protein is an attractive target for drug development. The first approved BRAF^V600E^ inhibitor vemurafenib (Figure 8) has demonstrated tumor regression in melanoma with a confirmed response rate of over 50% during phase III trials with eventual FDA approval in 2011 (Tsai et al., 2008; Bollag et al., 2012). Dabrafenib (2013) and encorafenib (2018) (Figure 8) are the other two approved BRAF^V600E^ inhibitors. One of the first proposed mechanisms of acquired resistance to vemurafenib is upregulation of CRAF to maintain MAPK pathway activation and allow tumor progression (Montagut et al., 2008). However, this upregulation has not been significantly observed in clinical analysis of patients resistant to the drug. Upregulations of other pathways, including the platelet-derived growth factor receptor (PDGFR) pathway and insulin-like growth factor 1 receptor (IGF1R) pathway, as well as upregulation of upstream RAS oncogenes and the BRAF oncogene itself have been noted in several preclinical and clinical samples analyzed, any of which may confer resistance against vemurafenib (Nazarian et al., 2010; Villanueva et al., 2010; Shi et al., 2012). One mechanism of particular note, however, is the modification of BRAF protein through incorrect mRNA splicing, resulting in a truncated isoform of protein that constitutively dimerizes into its active form to continue the pathway. This dimer, termed p61-BRAF^V600E^, is not affected by vemurafenib, and signaling occurs as needed for cancer progression (Poulikakos et al., 2011).

The prevalence of resistance to BRAF inhibitors sparked interest in PROTAC technology to degrade the protein entirely, and resulted in efficient and potent molecules selective for mutated BRAF proteins. Cullgen reported the first two BRAF-PROTACs, compounds 12 and 23 (Figure 8), constructed by linking vemurafenib and pan-RAF inhibitor BI882370 to a CRBN thalidomide ligand through an alkyl and a PEG linker, respectively (Han et al., 2020). Both PROTACs demonstrated dose-dependent degradation of BRAF^V600E^ and leaving BRAF^WT^ untouched in cells. Further, the degraders both show impaired melanoma cell growth in culture.

A second series of BRAF PROTACs were independently developed in the Therrien/Sicheri labs (Posternak et al., 2020). Their PROTACs were CRBN-recruiting and derived from two different linker tethering sites, each on dabrafenib and BI882370 that are similar to compound 23. The most potent PROTAC, P4B (Figure 8), displayed a DC_50_ of 12 nM and a D_max_ of 82% for BRAF^V600E^ in A375 cells and superior specificity and potency in pathway inhibition after a 24-h treatment compared to BI882370. They further demonstrated that P4B is also superior to inhibitors against cell lines with specific mutations that typically confer resistance to inhibitors, including NCI-H1666 cell line harboring the BRAF^G466V^ mutation and the A375-VR cell line harboring the BRAF^V600E^ with a tandem duplication of the kinase domain. Their work shows degradation by P4B and decreased cell proliferation in vemurafenib-resistant cell line A375, characterized by a homozygous BRAF^V600E^ mutation. However, when analyzing other specific lines with unique BRAF mutations, the compound showed no significant degradation or overall effect on cell viability (Posternak et al., 2020). These results suggest that P4B may be able to overcome cancers with BRAF mutations at the specific locus deemed suitable for degrader use, but is no more effective than inhibitors in cancer cells harboring other polymorphisms.

The Crews lab developed an optimized BRAF-PROTAC SJF-0628 that uses a vemurafenib-based ligand linked to a VHL E3 ligase ligand through a short and rigid piperazine linker (Figure 8) (Alabi et al., 2021). Initial *in cellulo* studies showed that SJF-0628 induces dose-dependent degradation of various BRAF mutants, while leaving BRAF^WT^, ARAF, and CRAF undisturbed despite the ability of warhead vemurafenib to potently bind all of these protein variants. The molecule successfully degraded the mutated BRAF protein within 4 h to near completion, with sustained degradation and diminished downstream ERK phosphorylation for up to 72 h. Even after washout, cells only showed 30% BRAF recovery in 24 h, highlighting the extended activity and catalytic effects of PROTAC treatment (Alabi et al., 2021). Of note is the ability of SJF-0628 to promote degradation of BRAF protein even in its truncated, dimerized form in various cell lines, overcoming vemurafenib resistance acquired through this mutation. This study demonstrated the potential of utilizing PROTAC technology to address drug resistance due to BRAF mutations.

### BTK-Targeted PROTACs to Address Drug Resistance Caused by Binding Site Mutation

**FIGURE 9:**
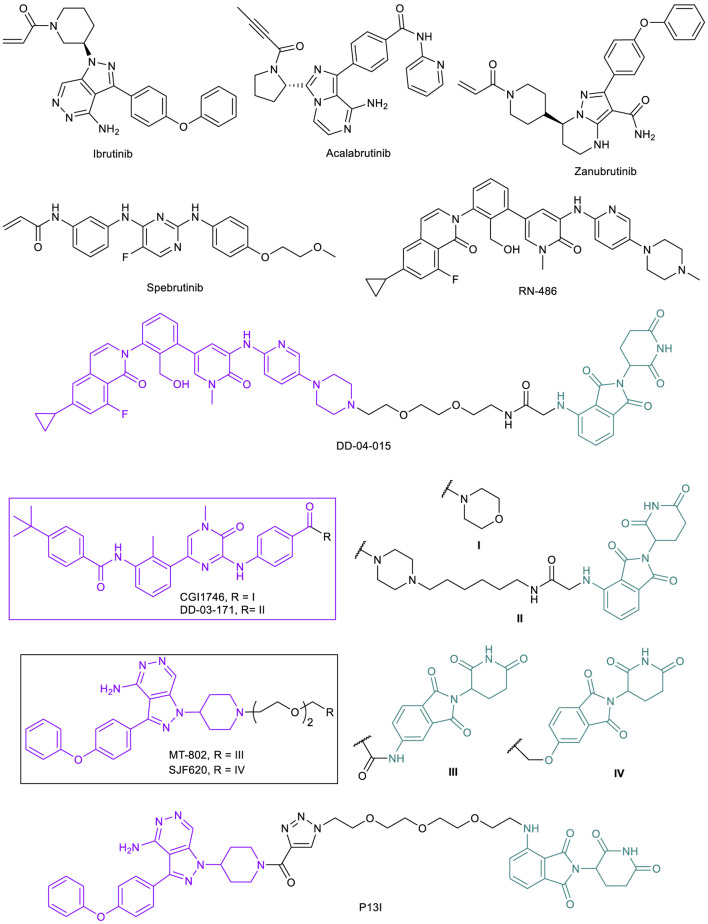
Key BTK small molecule inhibitors and BTK-targeted PROTACs.

B-cell lymphomas are often directly driven by chronic activation of B-cell receptor (BCR) and its mediator Bruton’s tyrosine kinase (BTK). Constitutive activation of BCR signaling *via* BTK is implicated in chronic lymphocytic leukemia (CLL), mantle cell lymphoma (MCL), diffuse large B cell lymphoma (DLBCL), as well as other malignancies (Valla et al., 2018). This uncontrolled activity results in cellular proliferation and subsequent B-lymphocyte malignancies in bone marrow, secondary lymphoid organs, and blood (Young et al., 2019). With the importance of BTK in cancer, early effort by Celera in search of inhibitors against this constitutive kinase activation resulted in the discovery of an irreversible covalent inhibitor later named ibrutinib (Figure 9). Unlike more mainstream noncovalent protein inhibitors, ibrutinib takes advantage of a reactive cysteine residue C481 within the BTK kinase domain, undergoing a Michael addition to the well-positioned acrylamide moiety on ibrutinib upon binding and irreversibly inhibiting the target. Initial studies showed significant potency, both *in vitro* and *in vivo*, along with selectivity for BTK over other related kinases (Pan et al., 2007). Ibrutinib was approved by the FDA in 2013 for the treatment of MCL and is now approved for multiple cancer indications. Acalabrutinib (2017; Figure 9) and zanubrutinib (2019; Figure 9), also targeting C481, are second-generation FDA approved irreversible BTK inhibitors with improved target selectivity (Wen et al., 2021).

Unfortunately, many patients receiving ibrutinib treatment experience disease relapse due to drug resistance. One study into BTK inhibition with ibrutinib in CLL samples explored efficacy of the drug *ex vivo*, and concluded that CLL cells with the 17p deletion (del17p) mutation along with TP53 mutations are substantially less sensitive to ibrutinib treatment than either alone, or no mutations at all (Amin et al., 2017). It remains unclear, however, what the cellular implications of these mutations are that allow cells to survive and proliferate even in the presence of a generally potent and effective inhibitor. Two other groups independently uncovered another resistance mechanism to covalent BTK inhibitors *via* the C481S mutation. Both groups highlight the fact that the C481S mutation was not present in baseline pretreatment samples, but are detected following ibrutinib therapy (Furman et al., 2014; Woyach et al., 2014). This mutation abolishes the covalent bond formation ability of ibrutinib and other irreversible BTK inhibitors with the protein, along with a potential steric clash, leading to insufficient inhibition and restored tumor growth.

To overcome the resistance caused by C481S mutation, two strategies have been proposed to directly target this mutation. One is to develop reversible BTK inhibitors that do not require C481 for strong binding. A number of reversible BTK inhibitors such as fenebrutinib, rilzabrutinib, spebrutinib, ARQ 531, and LOXO-305 are currently being evaluated in clinical trials (Estupinan et al., 2021). The second strategy is to develop BTK-targeted PROTACs (Arthur et al., 2020), and several groups have explored this option. Because irreversible binding to the POI will likely negate the catalytic nature of PROTACs, reported BTK-PROTACs are mostly based on non-covalent inhibitors or convert irreversible inhibitors to reversible covalent binders (Kiely-Collins, et al., 2021). The Gray lab reported the first CRBN-recruiting BTK-PROTAC DD-04-015, which is based on reversible BTK inhibitor RN486 (Figure 9) (Huang et al., 2018; Xu et al., 2012). This initial PROTAC demonstrated the validity of BTK degradation. In a follow up study, the Gray lab developed another CRBN-recruiting BTK-PROTAC DD-03-171, which is based on reversible BTK inhibitor CGI1746 (Figure 9) (Dobrovolsky et al., 2019). DD-03-171 is highly effective in degrading both BTK^WT^ and BTK^C481S^ that results in potent suppression of signaling and proliferation in cancer cells. Moreover, DD-03-171 was found to have more potent antiproliferative effects on MCL cells *in vitro* than inhibitors by degrading BTK and CRBN neo-substrates IKFZ1 and IKFZ3. DD-03-171 also displayed BTK degradation and tumor growth inhibition in *in vivo* PDX models (Dobrovolsky et al., 2019).

The Crews lab developed a novel CRBN-recruiting PROTAC, MT-802, based on a modified reversible ibrutinib scaffold and pomalidomide moiety (Figure 9). MT-802 displayed greater selectivity towards BTK when compared with ibrutinib. More importantly, MT-802 was able to induce potent degradation of both BTK^WT^ and BTK^C481S^ in samples from relapsed CLL patients with the C481S mutation, showing significantly lower phosphorylation levels (Buhimschi et al., 2018). Due to the catalytic nature of PROTACs, non-covalent binding of the warhead to BTK^C481S^, albeit weaker than irreversible covalent inhibitors, is sufficient to induce degradation of BTK in ibrutinib-resistant CLL. Based on MT-802, the Crews lab carried out a systematic medicinal chemistry campaign, which led to the identification of an equally potent analog SJF620 (Figure 9) with largely improved pharmacokinetic properties that are suitable for *in vivo* efficacy studies (Jaime-Figueroa et al., 2020).

The Rao lab independently developed BTK-PROTACs for the degradation of both BTK^WT^ and BTK^C481S^ (Sun et al., 2018). They surveyed the reversible version of ibrutinib and spebrutinib as a BTK-binding ligand and both CRBN ligand pomalidomide and MDM2 ligand RG-7112 as E3-binding ligand. P13I (Figure 9), which is based on ibrutinib and pomalidomide, was identified as a lead. The degrader also had no major off-target degradation, and was capable of targeting BTK proteins harboring the C481S mutation (Sun et al., 2018). These studies further confirmed the potential benefit of using PROTACs for relapsed disease caused by acquired mutations to inhibitors.

Further, Nurix has developed two orally bioavailable CRBN-recruiting BTK PROTACs: NX-2127 (NCT04830137) (with IMiD activity) and NX-5948 (NCT05131022) (lacking IMiD activity). Both have entered Phase I clinical trials (structures undisclosed) and have the ability to degrade clinically relevant BTK mutations that confer resistance to BTK inhibitors. Further, NX-5948 is capable of crossing the blood-brain barrier in animal models. (Robbins et al., 2020; Robbins et al., 2021). Though initial results are still forthcoming, the potential of these PROTACs in clinical trials is noteworthy.

## Discussion and Conclusion

Targeted cancer therapy has altered the way many types of cancer are treated over the last two decades, but the clinical utility of these cancer drugs is often limited by the seemingly inevitable development of drug resistance. With the understanding of the emerging mechanisms of resistance, subsequent generations of drugs could be developed to regain the treatment efficacy in patients. However, the cost and technical barrier to achieve the continued success in countering drug resistance may eventually make such practice impractical. Thus, there is an urgent need to develop novel treatment strategies and therapeutic modalities (Vasan et al., 2019). The advent of PROTAC technology has been paradigm-shifting in drug discovery, by offering many potential advantages over occupancy-driven protein inhibitors (Békés et al., 2022). PROTACs are being developed to target a number of clinically relevant targets and more than a dozen of them have been advanced to clinical trials (Mullard, 2021; Garber, 2022), demonstrating the great promise of this new therapeutic modality. As indicated in the examples summarized above, PROTACs offer an effective approach to address various forms of emerging drug resistance in response to SMIs. Consistent with their event-driven pharmacology, the catalytic removal of the target protein in its entirety and degradation driven by target binding rather than function disruption, PROTACs are well-suited for treatment of a number of clinically relevant, target therapy-induced resistance mechanisms. This includes 1) drug binding impaired by point mutations, 2) mutations resulting in target constitutive activation, 3) mutations inducing binding domain conformation changes, 4) gain of scaffolding function through target complex rearrangement, 5) target protein overexpression, 6) increased competition from endogenous ligands, and 7) splicing mutations. PROTACs can be constructed based on neutral binders that bind anywhere on a target protein, which opens up new opportunities to tackle mutation variants. Thus, in the situation that mutations cause a complete loss of binding affinity of existing ligands or loss of binding site, new binders could be potentially developed for PROTACs.

Overall, PROTACs appear to be promising in solving at least some of the challenges facing targeted therapies in the context of drug resistance. However, PROTAC technology is not without its potential limitations, and to the topic of this review, the possibility of cancer cells gaining resistance to PROTACs after extended treatment is a concern that has already attracted attention from academia and industry. The same mechanisms that allow for resistance to SMIs are possible for PROTACs, with alterations to either the POI or the E3 ligase that impair the ability of the PROTAC to engage ternary complex formation. However, the observed instances of drug induced resistance were not from mutations that affect the binding to the POI but occurred *via* genomic alterations that compromise the core components of the UPS (Mayor-Ruiz et al., 2019; Ottis et al., 2019; Zhang et al., 2019; Shirasaki et al., 2021). For example, BET-PROTACs ARV-771 (VHL-recruiting) and ARV-825 (CRBN-recruiting) were used in one experiment to produce resistant mutants through extended exposure to the OVCAR8 cell line, which was less sensitive to these PROTACs than other cells. These mutants were found to have mutations that affected the ubiquitination machinery, with different resistance origins based on what E3 ligase the PROTAC targeted (Zhang et al., 2019). ARV-711 caused mutations in the CUL2 gene, which is necessary for the interaction between the VHL subunit and the rest of the ubiquitin ligase complex (Zhang et al., 2019); further research using siRNA knockdown has shown that CUL2, RBX1, ELOB, ELOC, and the VHL subunit itself are all important proteins in the ubiquitin complex and thus vulnerable areas of resistance mutations for VHL-recruiting PROTACs (Ottis et al., 2019). ARV-825, on the other hand, was found to cause resistance through the deletion in the CRBN gene (Zhang et al., 2019), and siRNA also pointed to RBX1 and DDB1 as potential influential mutation sites for resistance (Ottis et al., 2019). Even after pausing and continuing treatment of cells with these PROTACs, cells were unaffected, indicating permanent genetic changes conferring resistance through mutation (Zhang et al., 2019). Unfortunately, these studies are currently limited as BRD4 is the only POI being investigated. Other resistance mechanisms are possible and should be considered for future development, such as resistance originating from the POI rather than the ubiquitin complexes, but further studies are needed to confirm the extent of effect from these mutations. As indirect evidence, studies on myeloma patient samples have found that the resistance to immunomodulatory imide drugs (IMiDs) such as pomalidomide and lenalidomide arise from genomic alterations in both the components of CRBN ligase machinery and the target proteins IKZF1/3 (Barrio et al., 2020; Gooding et al., 2021). These results also suggest that patients with prior exposure to IMiD treatments may have limited response to CRBN-based PROTACs. In another study, it was found that upregulation of USP15, a deubiquitinating enzyme (DUB), can antagonize IMiDs and CRBN-based PROTAC-induced ubiquitination, thereby preventing degradation, suggesting DUB overexpression is another potential resistance mechanism to PROTACs (Nguyen 2021).

Despite the possibility of eventual resistance occurring, PROTACs continue to increase in potential for effective treatments because of the advantages they offer over conventional SMIs, especially as these inhibitors can quickly give rise to resistant tumors. However, it is still too early to know if these promises can be translated into clinical benefits. With many clinical trials ongoing and new compounds entering clinical testing, we will soon see proof-of-concept data emerging from these studies. With our improved ability to predict potential drug resistance mechanisms ahead of real occurrence in clinical settings, it is possible to design strategies to prevent, slow down, or overcome such resistance. To this end, PROTACs could have an advantage in terms of overcoming their own resistance, as it has been shown that resistance to a VHL-recruiting PROTAC does not necessarily translate to resistance against a CRBN-recruiting PROTAC, or vice versa (Zhang et al., 2019; Shirasaki et al., 2021). With over 600 E3 ligases in the human genome, new E3 ligases and ligands that are suitable for PROTAC design will be identified. A library of E3 recruiters could be used with variation to continue treatments and lower chances of resistance, resulting in further generations that are more potent and efficacious in treating various cancers.

## Abbreviations

AR, androgen receptor; AURKA, aurora kinase A; BCR, breakpoint cluster region; BET, bromodomain and extraterminal protein; BRD4, bromodomain-containing protein 4; BTK, Bruton’s tyrosine kinase; CLL, chronic lymphocytic leukemia; CDK (1, 2, etc), cyclin-dependent kinases; CRBN, cereblon; CML, chronic myeloid leukemia; CRPC, castration-resistant prostate cancer; CYP1B1, cytochrome p450 1B1; c-MYC, cellular myelocytomatosis oncogene; DLBCL, diffuse large B cell lymphoma; EGFR, epidermal growth factor receptor; ER, estrogen receptor; ERK, extracellular signal-regulated kinase; IAP, inhibitor of apoptosis protein; IGF1R, insulin-like growth factor 1 receptor; MAPK, mitogen-activated protein kinase; MCL, mantle cell lymphoma; NSCLC, non-small cell lung cancer; PROTAC, proteolysis targeting chimera; POI, protein of interest; TKI, tyrosine kinase inhibitors; PSA, prostate-specific antigen; RAS, rat sarcoma virus oncogene; SERMs, selective estrogen receptor modifiers; SERDs, selective estrogen receptor degraders; TKIs, tyrosine kinase inhibitors; siRNA, small interfering RNA; PARP, poly (ADP-ribose) polymerase; RAF, rapidly accelerated fibrosarcoma; PDGFR, platelet-derived growth factor receptor; PEG, polyethylene glycol; PTK2B, protein tyrosine kinase two beta; SNIPER, specific and non-genetic inhibitor of apoptosis protein-dependent protein eraser; VHL, von Hippel-Lindau.
